# Supplementary material for: Development and validation of a dual language needs assessment tool for people living with colorectal cancer (NeAT-CC)
Source: PLoS One. 2025 Oct 21;20(10):e0332930. doi: 10.1371/journal.pone.0332930 (PMC12539725; doi:10.1371/journal.pone.0332930)
Supplement: S1 Table — This table presents the test–retest reliability analysis for the Needs Assessment Tool for Colorectal Cancer (NeAT-CC), including Weighted Kappa for each item. (DOCX) [file pone.0332930.s001.docx]

**Additional files Supplementary Table**

i) File name: **S1 Table. Test-retest reliability results of the Needs Assessment Tool for Colorectal Cancer (NeAT-CC).**

File format: Doc

Description of data: This table presents the test–retest reliability analysis for the Needs Assessment Tool for Colorectal Cancer (NeAT-CC), including Weighted Kappa for each item.

S1 Table. Test-retest reliability result

| **Initial domain**  **/Item** | **Question** | **Weighted Kappa** | **95%CI** | **Agreement level** |
| --- | --- | --- | --- | --- |
| **A) Diagnosis** | | | | |
| A1 | I need healthcare professionals to be understanding and sensitive to my feelings when breaking the news that I have cancer | 0.5143 | 0.241 to 0.787 | Moderate |
| A2 | I need access to counseling and psychological services as soon as possible after the doctor tells me that I have cancer | 0.5576 | 0.335 to 0.780 | Moderate |
| A3 | I need to be informed about cancer support groups as soon as possible | 0.5283 | 0.282 to 0.775 | Moderate |
| **B) Psychosocial** | | | | |
| B4 | I need healthcare professionals to be more compassionate during my treatment sessions and follow-ups | 0.5431 | 0.349 to 0.737 | Moderate |
| B5 | I need access to counseling and psychological services | 0.6414 | 0.479 to 0.803 | Substantial |
| B6 | I need religious or spiritual support to cope with my emotions | 0.4199 | 0.131 to 0.709 | Moderate |
| B7 | I need help to cope with my worries and fear of recurrence | 0.5439 | 0.380 to 0.708 | Moderate |
| B8 | I need help to accept changes in my body (example: surgery scars, wearing a colostomy bag) and to boost my body image and self confidence | 0.4874 | 0.243 to 0.732 | Moderate |
| B9 | I need my family to give me more emotional support | 0.6642 | 0.438 to 0.891 | Substantial |
| B10 | I need my close family members to receive emotional/psychological support | 0.596 | 0.333 to 0.859 | Moderate |
| B11 | I need to deal with discrimination in social settings because of my cancer | 0.5189 | 0.296 to 0.742 | Moderate |
| B12 | I need to join a cancer support group | 0.4675 | 0.227 to 0.708 | Moderate |

**S1 Table.**  **Continued**

| **Initial domain**  **/Item** | **Question** | **Weighted Kappa** | **95%CI** | **Agreement level** |
| --- | --- | --- | --- | --- |
| **C) Information** | | | | |
| C13 | I need the information that the doctor gives me to be easily understood | 0.4850 | 0.251 to 0.719 | Moderate |
| C14 | I need the doctor to give me written information about my cancer to read later on | 0.4527 | 0.156 to 0.749 | Moderate |
| C15 | I need adequate information about all the different treatment options before I choose to have them | 0.4248 | 0.104 to 0.745 | Moderate |
| C16 | I need to be informed about my test results as soon as they are known | 0.5033 | 0.190 to 0.817 | Moderate |
| C17 | I need more cancer-related materials (example: books, brochures, videos, etc.) in the waiting area or online at the hospital website. | 0.4699 | 0.243 to 0.697 | Moderate |
| C18 | I need my spouse/family to be given information regarding my cancer | 0.4890 | 0.173 to 0.805 | Moderate |
| C19 | I need information on things that I can do to take better care of myself | 0.5101 | 0.280 to 0.741 | Moderate |
| C20 | I need information about diet to know what I should avoid or what should I be eating more | 0.4778 | 0.177 to 0.778 | Moderate |
| C21 | I need my oncologist to openly discuss traditional and complementary medicine with me | 0.6240 | 0.404 to 0.844 | Substantial |
| C22 | I need the hospital to give traditional and complementary medicine services along with conventional treatment | 0.4492 | 0.226 to 0.673 | Moderate |
| C23 | I need information and help in coping with my sexual difficulties | 0.5094 | 0.290 to 0.729 | Moderate |
| C24 | I need to be informed about fertility issues, and potential solutions, before my treatment | 0.5882 | 0.406 to 0.770 | Moderate |

**S1 Table.** **Continued**

| **Initial domain**  **/Item** | **Question** | **Weighted Kappa** | **95%CI** | **Agreement level** |
| --- | --- | --- | --- | --- |
| **D) Practical** | | | | |
| D25 | I need to be seen by the same team of doctors at every appointment | 0.4627 | 0.230 to 0.696 | Moderate |
| D26 | I need the waiting time in the hospital to be shortened | 0.4848 | 0.292 to 0.678 | Moderate |
| D27 | I need to be given an explanation if there is a delay in the doctor attending to me | 0.4239 | 0.165 to 0.683 | Moderate |
| D28 | I need help in making appointments and someone to call if I need to change appointments | 0.5602 | 0.317 to 0.803 | Moderate |
| D29 | I need all my hospital appointments to be set on the same day whenever possible | 0.4549 | 0.231 to 0.679 | Moderate |
| D30 | I need to know who to contact if I have any questions or concerns on my disease or treatment in between hospital appointments | 0.6199 | 0.393 to 0.847 | Substantial |
| D31 | I need the hospital facilities and surroundings to be clean, comfortable and pleasant | 0.8109 | 0.631 to 0.991 | Substantial |
| D32 | I need the hospital facilities to be located near each other (example: pharmacy, clinic, payment counter, wards) | 0.5484 | 0.277 to 0.820 | Moderate |
| D33 | I need reserved parking spaces specifically for cancer patients who self-drive to the hospital | 0.5648 | 0.340 to 0.789 | Moderate |
| D34 | I need my doctor to manage better the side effects of my cancer and treatments | 0.4670 | 0.203 to 0.731 | Moderate |
| D35 | I need my family doctor/ GP to be also knowledgeable about the side effects of my cancer treatment | 0.6144 | 0.394 to 0.835 | Substantial |
| D36 | I need help to cope with limitations in daily activities/activities that I used to do | 0.5709 | 0.321 to 0.821 | Moderate |
| D37 | I need help to care for my family members who are depending on me (example: children, parents, spouses) | 0.6556 | 0.336to 0.975 | Substantial |

**S1 Table**. **Continued**

| **Initial domain**  **/Item** | **Question** | **Weighted Kappa** | **95%CI** | **Agreement level** |
| --- | --- | --- | --- | --- |
| **E) Financial** | | | | |
| E38 | I need information on the costs of my treatments before starting them | 0.6089 | 0.408 to 0.810 | Substantial |
| E39 | I need assistance to pay for my cancer treatments | 0.6565 | 0.439 to 0.873 | Substantial |
| E40 | I need help to understand my insurance benefits and coverage, and in making claims | 0.8712 | 0.778 to 0.964 | Substantial |
| E41 | I need to buy health insurance after my cancer diagnosis | 0.7222 | 0.559 to 0.886 | Substantial |
| E42 | I need guidance and assistance in obtaining financial assistance | 0.5279 | 0.273to 0.783 | Moderate |
| E43 | I need affordable parking and transportation when I come to the hospital | 0.5181 | 0.273 to 0.763 | Moderate |
| E44 | I need affordable colostomy bags, diapers or wigs | 0.5583 | 0.337 to 0.779 | Moderate |
| E45 | I need help to pay for dietary supplements (example: special milk, special food) | 0.5223 | 0.299 to 0.745 | Moderate |
| E46 | I need affordable special equipment (example: wheelchair, special bed) | 0.6095 | 0.403 to 0.816 | Substantial |
| E47 | I need to pay for hired help at home (example: maid, babysitter) | 0.5161 | 0.332 to 0.700 | Moderate |
| E48 | I need help to cope with a reduced household income due to my illness | 0.5788 | 0.347 to 0.810 | Moderate |
| **F) Employment (n=14)** | | | | |
| F49 | I need discrimination at my workplace to be addressed (example: promotion, included in important projects, having job security) | 0.7742 | 0.573 to 0.976 | Substantial |
| F50 | I need workplace flexibility (example: time off for hospital appointments, changes in job scope, flexible hours, work from home,) | 0.8250 | 0.655 to 0.995 | Substantial |
| F51 | I need help to find a new job after my cancer diagnosis | 0.5950 | 0.345 to 0.845 | Moderate |
