# Supplementary material for: Development and validation of a dual language needs assessment tool for people living with colorectal cancer (NeAT-CC)
Source: PLoS One. 2025 Oct 21;20(10):e0332930. doi: 10.1371/journal.pone.0332930 (PMC12539725; doi:10.1371/journal.pone.0332930)
Supplement: S2 Table — This table presents factor loadings for each item across the six domains. (DOCX) [file pone.0332930.s002.docx]

**Additional files Supplementary Table**

File name: **S2 Table. Confirmatory factor analysis of the needs assessment tool for colorectal cancer (NeAT-CC).**

File format: Doc

Description of data: This table presents factor loadings for each item across the six domains.

**S2 Table. Confirmatory factor analysis of the needs assessment tool for colorectal cancer (NeAT-CC)**

| **Domain** | **Items** | | **Diagnosis need** | **Psychosocial and information need** | **Healthcare need** | **Practical and living with cancer** | **Financial need** | **Employment needs** |
| --- | --- | --- | --- | --- | --- | --- | --- | --- |
| **Diagnosis** | A1 | Understanding healthcare  professional | 0.724 |  |  |  |  |  |
|  | A2 | Access to counselling | 0.870 |  |  |  |  |  |
|  | A3 | Cancer Support | 0.894 |  |  |  |  |  |
| **Psychosocial**  **and**  **information** | B4 | Compassionate healthcare  professional |  | 0.766 |  |  |  |  |
|  | B5 | Access to counselling |  | 0.730 |  |  |  |  |
|  | B6 | Spiritual/religious support |  | 0.739 |  |  |  |  |
|  | B7 | Help to cope with worries |  | 0.755 |  |  |  |  |
|  | B8 | Help to accept body |  | 0.764 |  |  |  |  |
|  | B9 | Family support |  | 0.660 |  |  |  |  |
|  | B10 | Family to receive support |  | 0.505 |  |  |  |  |
|  | B11 | Deal with discrimination |  | 0.563 |  |  |  |  |
|  | B12 | Join support group |  | 0.608 |  |  |  |  |
|  | C13 | Information easily understood |  | 0.665 |  |  |  |  |
|  | C16 | Test result |  | 0.600 |  |  |  |  |
|  | C17 | More reading materials |  | 0.606 |  |  |  |  |
|  | C18 | Family to be given information |  | 0.672 |  |  |  |  |
|  | C19 | Information for better care |  | 0.641 |  |  |  |  |
|  | C20 | Information on diet |  | 0.602 |  |  |  |  |
| **Healthcare** | D25 | Seen by same doctor/team |  |  | 0.670 |  |  |  |
|  | D26 | Waiting time to be shorten |  |  | 0.665 |  |  |  |
|  | D27 | Explanation if delay |  |  | 0.591 |  |  |  |
|  | D28 | Help with appointment/call |  |  | 0.722 |  |  |  |
|  | D29 | Appointment on the same day |  |  | 0.644 |  |  |  |
|  | D30 | Contact for questions |  |  | 0.754 |  |  |  |
|  | D31 | Hospital facilities to be clean |  |  | 0.668 |  |  |  |
|  | D32 | Near hospital facilities |  |  | 0.655 |  |  |  |
|  | D33 | Reserved parking |  |  | 0.589 |  |  |  |
|  | D34 | Doctor to manage side effects |  |  | 0.704 |  |  |  |
|  | D35 | GP to be knowledgeable |  |  | 0.680 |  |  |  |
|  | E43 | Affordable parking |  |  | 0.630 |  |  |  |

**S2 Table. Continued**

| **Domain** | **Items** | | **Diagnosis need** | **Psychosocial and information need** | **Healthcare need** | **Practical and living with cancer** | **Financial need** | **Employment needs** |
| --- | --- | --- | --- | --- | --- | --- | --- | --- |
| **Practical and**  **living with**  **cancer**  **needs** | C21 | Discuss traditional/  complementary medicine |  |  |  | 0.698 |  |  |
|  | C22 | Given traditional/  complementary medicine |  |  |  | 0.699 |  |  |
|  | C23 | Information on sexual |  |  |  | 0.733 |  |  |
|  | C24 | Information on fertility |  |  |  | 0.650 |  |  |
|  | D36 | Cope with daily activities |  |  |  | 0.562 |  |  |
|  | D37 | Care for dependent |  |  |  | 0.581 |  |  |
|  | E40 | Understand insurance benefit |  |  |  | 0.645 |  |  |
|  | E41 | Buy insurance after diagnosis |  |  |  | 0.530 |  |  |
| **Financial** | E39 | Assistance to pay for treatment |  |  |  |  | 0.748 |  |
|  | E42 | Guidance in obtaining  financial assistance |  |  |  |  | 0.803 |  |
|  | E44 | Affordable colostomy, diapers |  |  |  |  | 0.626 |  |
|  | E45 | Help to pay dietary supplements |  |  |  |  | 0.859 |  |
|  | E46 | Affordable equipment |  |  |  |  | 0.775 |  |
|  | E47 | Need to hired help |  |  |  |  | 0.611 |  |
|  | E48 | Help cope with reduce income |  |  |  |  | 0.854 |  |
| **Employment** | F49 | Need discrimination at workplace  to be addressed |  |  |  |  |  | 0.809 |
|  | F50 | Need workplace flexibility |  |  |  |  |  | 0.864 |
|  | F51 | Need to find new job after  cancer diagnosis |  |  |  |  |  | 0.813 |
