# Supplementary material for: Development and validation of a dual language needs assessment tool for people living with colorectal cancer (NeAT-CC)
Source: PLoS One. 2025 Oct 21;20(10):e0332930. doi: 10.1371/journal.pone.0332930 (PMC12539725; doi:10.1371/journal.pone.0332930)
Supplement: S3 Table — This table displays the HTMT ratios between domains of the NeAT-CC to demonstrate discriminant validity. (DOCX) [file pone.0332930.s003.docx]

**Additional files Supplementary Table**

File name: **S3Table. Heterotrait–monotrait (HTMT) ratios of the domains for discriminant validity.**

File format: Doc

This table displays the HTMT ratios between domains of the NeAT-CC to demonstrate discriminant validity.

**S3 Table. HTMT ratios of the domains for discriminant validity**

|  | **Diagnosis need** | **Employment** | **Financial need** | **Healthcare need** | **Practical and living with cancer** | **Psychosocial and information** |
| --- | --- | --- | --- | --- | --- | --- |
| **Diagnosis need** |  |  |  |  |  |  |
| **Employment** | 0.311 |  |  |  |  |  |
| **Financial need** | 0.505 | 0.507 |  |  |  |  |
| **Healthcare need** | 0.575 | 0.271 | 0.473 |  |  |  |
| **Practical and living with cancer** | 0.651 | 0.54 | 0.73 | 0.531 |  |  |
| **Psychosocial and information need** | 0.869 | 0.318 | 0.573 | 0.725 | 0.808 |  |
