# Supplementary material for: Development and validation of a dual language needs assessment tool for people living with colorectal cancer (NeAT-CC)
Source: PLoS One. 2025 Oct 21;20(10):e0332930. doi: 10.1371/journal.pone.0332930 (PMC12539725; doi:10.1371/journal.pone.0332930)
Supplement: Attachment — Submitted filename: PeerReview_Bujang_PONE-D-24-35504_NeAT-CC.docx [file pone.0332930.s004.docx]

Thank you for the opportunity to provide peer review for the article “Development and validation of a dual language needs assessment tool for people living with colorectal cancer (NeAT-CC)”.

The well written article focuses on creating a dual language needs assessment tool for people living with or after colorectal cancer in a multi-ethnic and resource limited country. The authors argue that previous needs assessment tools were mostly developed for affluent countries and that so far there are no tools specifically validated for colorectal cancer in low- to mid-income countries. To create their needs assessment tool, the authors followed rigorous methodological guidelines. This includes the COnsensus-based Standards for the selection of health status Measurement INstruments (COSMIN) checklist, validations with factor analyses and a later correlation of the NeAT-CC with known quality of life questionnaires.

Major points:

- Statistics: Did you test for other factor models for the confirmatory factor analysis (CFA)?
- Are there any specific plans in the future with the newly validated NeAT-CC tool, how you would bring it into practice, who could be analysing the questionnaires and implement the changes? Are there any cut-offs of the patient scores that help with its interpretation?

Minor points:

- Please state more in detail, why other already available assessment tools are not applicable in Malaysia. Where are the differences (better or worse)? What exactly is missing in the other tools and which problems would arise if the other tools are applied in your population? Could the NeAT-CC tool instead be used also for high-income countries? Or if not, what are the possible problems when applying the NeAT-CC in those countries.
- In the results of the abstract it is not clear which are the six domains of needs. *“(…) namely diagnosis, psychosocial and information, healthcare, practical and living with cancer, financial and employment.”*
- Is the study population representative of the general population? In 2.2.1 it is described, that some people were excluded that were unfit or couldn’t converse (maybe add a flow-chart to clarify). There should be more information if the baseline characteristics of the studied patients match with the general population of Malaysia (percentage of private insurance, income, etc. as in Table 2).
- Abbreviations should be stated in every Table.
- It would be interesting to have a longitudinal study to see, whether the data are reproducible over time and if the NeAT-CC is able to show changes in needs of colorectal cancer patients over time. This not only for the individual itself but also generally for the whole population, if new services and programs are implemented based on the needs evaluations with the NeAT-CC.
- I could not read Figure 1.

The study uses robust statistics to address an important area. It can, with some refinements, potentially make a significant impact in the field of survivorship care.
